# A new circa 2007 biomass map for China differs significantly from existing maps

**DOI:** 10.1038/s41597-024-03092-8

**Published:** 2024-03-11

**Authors:** Wenquan Dong, Edward T. A. Mitchard, Maurizio Santoro, Man Chen, Charlotte E. Wheeler

**Affiliations:** 1 School of GeoSciences, University of Edinburgh, Edinburgh, EH9 3FF UK; 2 GAMMA Remote Sensing, 3073 Gümligen, Switzerland; 3 Department of Plant Sciences and Conservation Research Institute, University of Cambridge, Cambridge, CB2 3EA UK

**Keywords:** Carbon cycle, Forest ecology, Forestry

## Abstract

The forest area of China is the fifth largest of any country, and unlike in many other countries, in recent decades its area has been increasing. However, there are substantial differences in estimates of the amount of carbon this forest contains, ranging from 3.92 to 17.02 Pg C for circa 2007. This makes it unclear how the changes in China’s forest area contribute to the global carbon cycle. We generate a circa 2007 aboveground biomass (AGB) map at a resolution of 50 m using optical, radar and LiDAR satellite data. Our estimates of total carbon stored in the forest in China was 9.52 Pg C, with an average forest AGB of 104 Mg ha^−1^. Compared with three existing AGB maps, our AGB map showed better correlation with a distributed set of forest inventory plots. In addition, our high resolution AGB map provided more details on spatial distribution of forest AGB, and is likely to help understand the carbon storage changes in China’s forest.

## Background & Summary

Forests reduce the impact of climate change, which is the greatest environmental challenge of the 21st century^1^, by capturing CO_2_ from the atmosphere, and acting as guardians of a carbon store in their wood, roots, and soils. However, there are major uncertainties as to the amount of carbon stored in forests, and how this is changing through time^2^. The carbon stored in forests changes as a combination of the processes of deforestation, degradation and growth driven by both natural and anthropogenic disturbances, and climate change itself. This leads to fluxes in stored carbon, making forest carbon stock one of the greatest sources of uncertainty in the global carbon cycle^2–4^. It is important to reduce these uncertainties to improve our understanding of the carbon cycle and thus various elements of Earth system and climate models. This reduction is also crucial to enable policies to conserve and increase forest carbon storage to be designed and monitored.

Modern remote sensing data, cloud computing, and machine learning capacity, enable the production of high quality maps of AGB, the largest carbon pool in most forests, and a carbon pool that can change substantially over time^5^. This increasing capacity to generate high quality maps of AGB has been shown by the recent release of a large number of global products^6–9^. However, such global products can have high uncertainties at a local or regional level, and often perform poorly when compared to field plots^9–11^. Given this, there is clearly a place for regional or country-specific maps where datasets and methods are tuned to local conditions, and that perform well against independent validation datasets^12^.

China covers 6.3% of the world’s land surface, has 18% of its people (The World Bank, 2021), and an estimated 5.4% of its forest^13^. As forests play such an important role in the global climate system and in terrestrial ecosystems, what is happening in China’s forests is important to the global climate system. The area of China’s forest was 102 million hectares (ha) in 1949, and it remained at 106 million ha until 1994–1998^14^, and then increased dramatically to 220 million ha in 2020, accounting for 23% of China’s area^13^. Typically, substantial increases in forest area lead to increases in total AGB. However in China’s case, it is possible that higher-biomass, old-growth forests are being replaced with new, lower-biomass forests or plantations^15,16^.

Currently, information of forest AGB can be derived from field inventory data and Earth Observation (EO) data. Conventionally, forest AGB estimation requires inventory forest variables such as tree species, diameter at breast height (DBH), and tree height, measured in fixed area forest inventory plots, which are converted into estimates of tree biomass through allometric equations^17^. Although conventional inventory techniques generally provide accurate estimates of forest AGB at plot level^18^, it is time-consuming, only possible to collect data covering a very small area, requiring good statistical stratification and placement of a large number of field plots in order for them to represent the wider forest, and sometimes impractical due to inaccessibility. It is obviously impossible in the present time to add sampling plots or locations retroactively for historical data collection. On the other hand, EO data, from satellites or aircraft, has shown promising capabilities for consistent and systematic observations of the dynamics of forest ecosystems^19–22^. However, producing accurate EO-based results relies on consideration of local factors, ideally using local field calibration data as well as appropriate EO data sources.

Some studies of AGB in China have been conducted using satellite data. However, these studies produce very different estimates, due to differences in their prediction models and dataset sources (Table 1). The total carbon estimates from the map of Su^23^ are four times higher than that of GEOCARBON^6^. While these maps use different forest cover maps to mask non-forest areas, resulting in variations in forest area, and the maps are for slightly different periods, this disparity in area and time cannot explain much of the observed difference.

**Table 1:** Total carbon stored in forest AGB in China from a number of studies, along with the area covered by the maps.

Source	Carbon (Pg C)	Period	Area
GEOCARBON^6^	3.92	2000–2010	Global
Huang, *et al*.^56^	5.44	2006	China
CCI Biomass, v3^9^	5.94	2010	Global
Yin, *et al*.^70^	8.56	2001–2013	China
Li, *et al*.^71^	9.87	2010	China
Su, *et al*.^23^	17.02	2004	China

The carbon estimated for GEOCARBON, CCI Biomass, V3 and the Su maps were derived using AGB maps provided by the respective studies. For GEOCARBON and CCI Biomass, V3, non-forested areas were excluded based on Hansen forest cover data^35^. The Su map had already excluded non-forest areas in their AGB map. Estimates for all other sources were obtained directly from the respective publications.

Therefore, our study aims to produce a reliable 2007 AGB map for China. We chose to map 2007 for a number of reasons, summarised as (i) it is a useful baseline year to compare against forest policy and (ii) a variety of remote sensing and field validation datasets is available around that date. The Asia-Pacific Network for Sustainable Forest Management and Rehabilitation (APFNet) was proposed in 2007, and it further marks the acceleration of the China’s reforestation/afforestation programmes, as well as the implementation of forest protection laws^24^. From the remote sensing standpoint, 2007 is where spaceborne LiDAR and suitable optical and radar satellite data are available. LiDAR data from satellites provide a direct estimate of height, which is closely related to biomass, unlike other satellite sensors which rely on indirect estimation, with the shape of relationships often varying from place to place^25^. The Ice, Cloud, and land Elevation Satellite (ICESAT) Geoscience Laser Altimeter System (GLAS) operated throughout the 2000s, including collecting lots of data in 2007, and provides a large number of ~65 m circular footprints with estimates of height, used to train and test the wall-to-wall maps of AGB we produce. In order to extrapolate across the landscape, we need Synthetic Aperture Radar (SAR) and optical datasets. The C-band Environmental Satellite (Envisat) Advanced Synthetic Aperture Radar (ASAR) and Advanced Land Observing Satellite (ALOS) Phased Array L-band SAR (PALSAR) were available around 2007. C-band SAR is capable of penetrating into the canopy, and L-band SAR can penetrate deeper into the canopy and thus obtain information of larger element of trees such stems^26^. Landsat-5 TM data was also used in the prediction, as optical data can capture detailed spectral information of forest canopy.

In this study, we introduced a new 2007 AGB map for China using high resolution multisource remote sensing data. Our model was trained specifically for China, considering its unique allometric equation and terrain factors. This dataset can serve as the historical baseline dataset for forest AGB change studies, as it outperforms existing maps when compared to two unseen field validation datasets.

## Methods

**Fig. 1:**
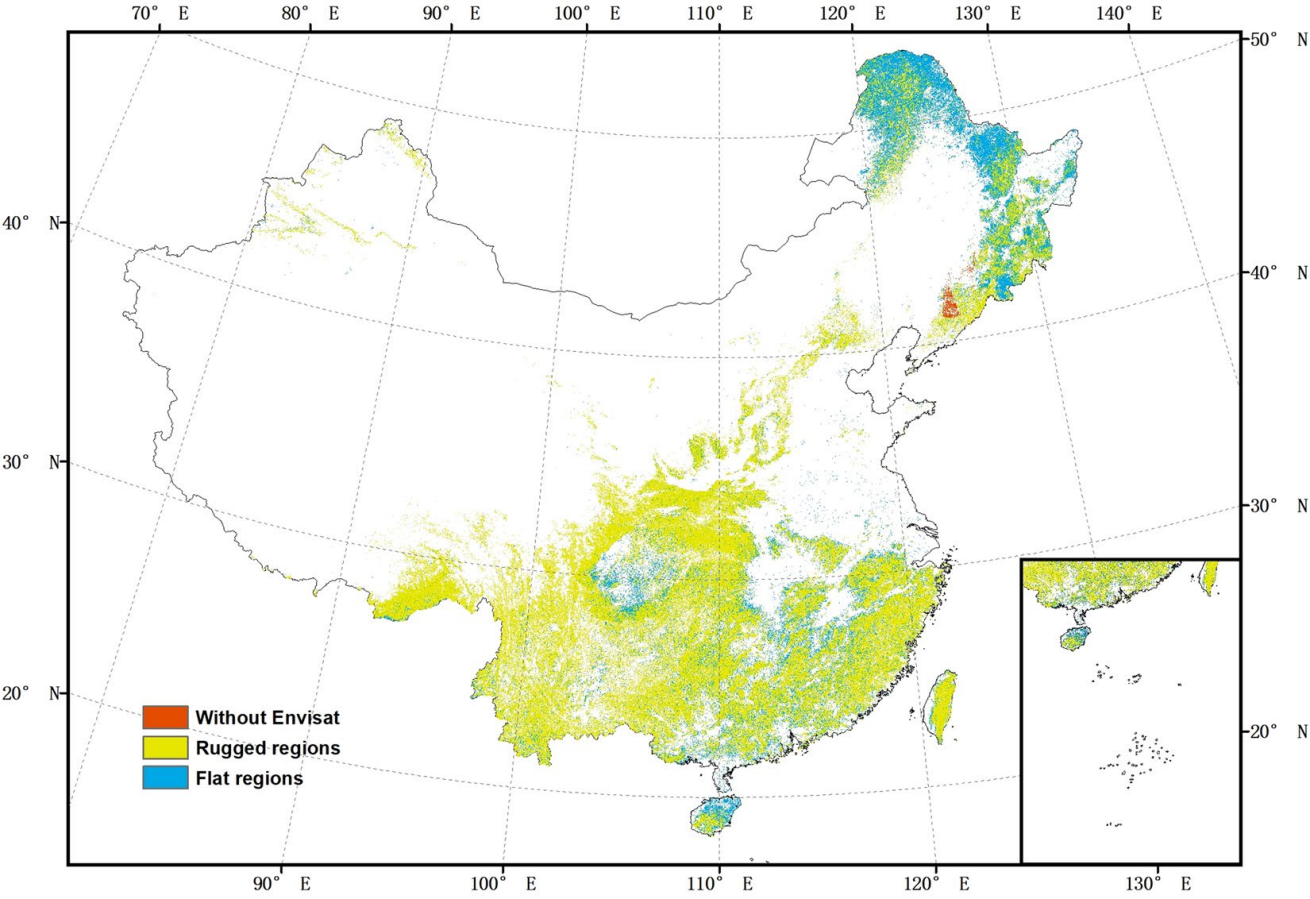
The spatial distribution of the three forested regions.

**Fig. 2:**
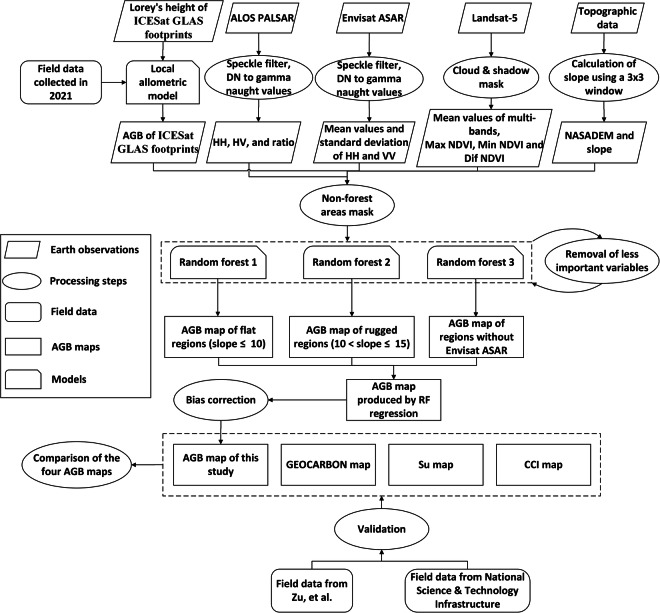
Overall workflow of forest AGB estimation and validation.

We generated point estimates of AGB from ICESat GLAS Lorey’s height data^27^, using local allometric relationships developed independently for northern and southern China using field data we collected in 40 forest inventory plots in China. We then predict AGB spatially to create a wall-to-wall map at a resolution of 50 m using a random forest model with L-band SAR from ALOS PALSAR, C-band SAR from Envisat ASAR, and optical satellite data from Landsat-5, all acquired around 2007, in addition to a Digital Elevation Model from the Shuttle Radar Topography Mission (SRTM) (collected in 2000). Both forest structure and remote sensing responses are different in sloped compared to flat areas^28,29^, so we generated continuous AGB maps for flat regions and rugged regions separately, to enable different prediction models for the two and thus reduce the errors in AGB estimates. Due to gaps in the coverage by Envisat ASAR (1.2% of China was missed), we generated a second AGB map with slightly lower accuracy to use in the regions without Envisat ASAR data (Fig. 1). As a last step, we combined the maps for flat region (slope ≤10°), rugged region (slope >10°) and region without Envisat data, to produce a final map for validation (Fig. 2).

Further detail about all these steps is given below, and code is available.

### Field data

#### Field data collected in 2021

**Fig. 3:**
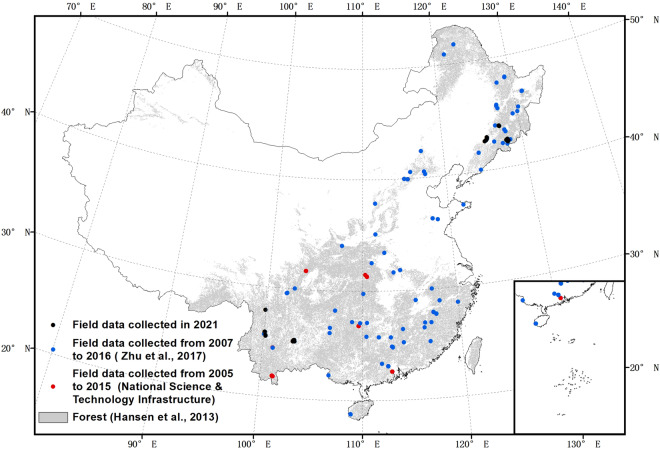
Spatial distribution of field data collected for this study in 2021 and compiled from other studies from 2005 to 2015.

26 field plots in northeastern China and 14 field plots in Southwestern China were measured in 2021 (Fig. 3). Each of the field plot was circular with a radius of 12.5 m (~0.05 ha). We measured tree height using Vertex IV and Transponder T3, and measured DBH of all the trees with DBH ≥5 cm. These measurements were used to estimate the AGB of each tree, utilizing the following biomass equation for China^30^:

1 $$W=0.1355\times {\left({D}^{2}H\right)}^{0.817}$$

Where, *W* is the AGB of each tree (kg), *D* is DBH (cm) and H is height of the tree (m). We summed the AGB of every tree to estimate total AGB per plot, and converted the total biomass to AGB density at the 1 ha scale (Mg ha^−1^).

We also calculated Lorey’s height of each plot using Eq. (2). Lorey’s height is an index of height whereby individual trees are weighted in proportion to their basal area, which enhances the significance of larger trees in the forest, and is closely correlated with AGB^25,31,32^.

2 $$Lorey=\frac{{\sum }_{i=1}^{N}B{A}_{i}{h}_{i}}{{\sum }_{i=1}^{N}B{A}_{i}}$$

where, *BA*_*i*_ represents the basal areas, and *h*_*i*_ represents the canopy height of the individual trees.

**Fig. 4:**
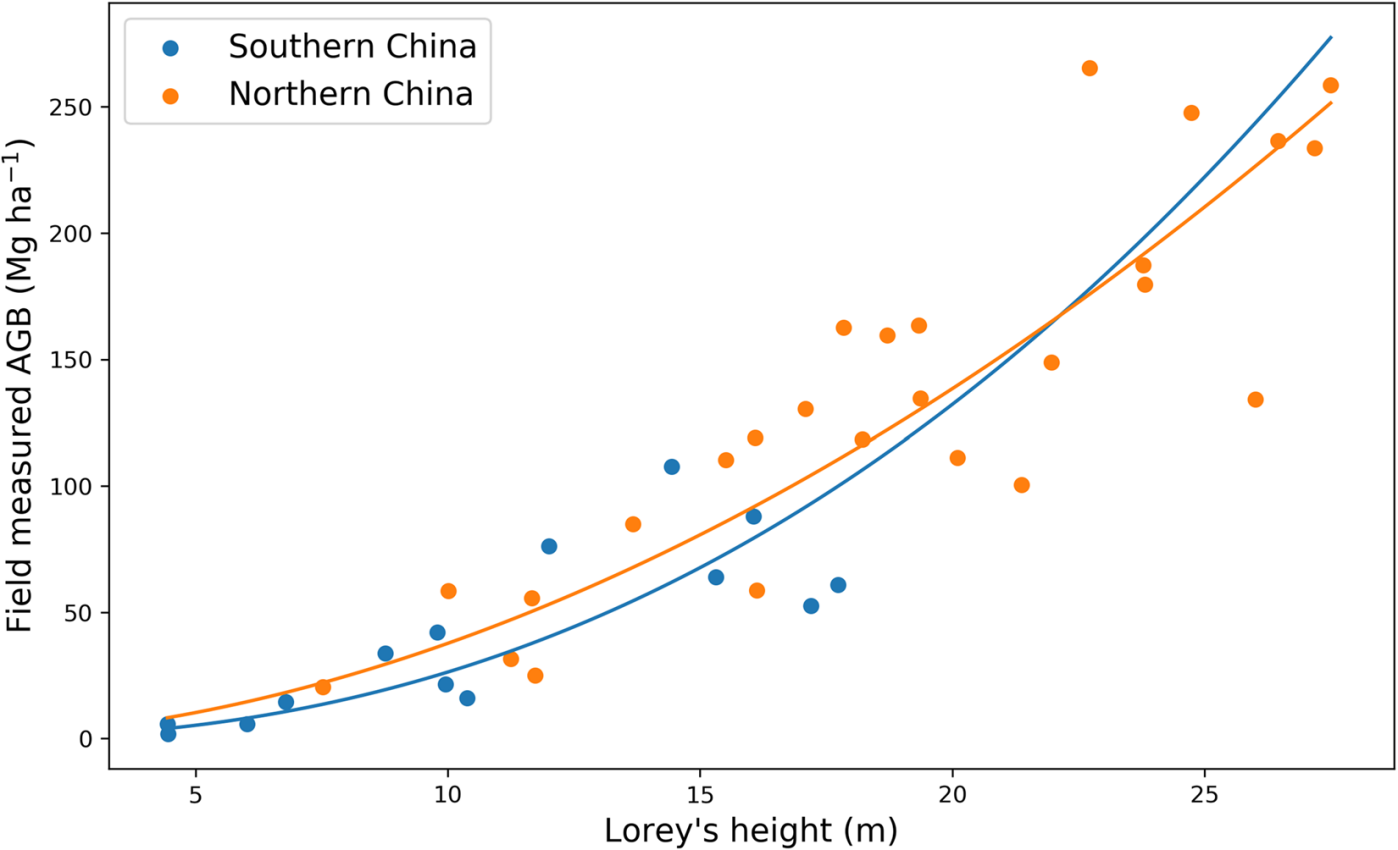
Allometric equations between field measured Lorey’s height and AGB. The solid lines are fitted lines. Both fitted lines were extended to the full range to provide a more comprehensive representation of the potential trends of the allometric equations.

We used these plot-level measurements to develop allometric equations between the field measured Lorey’s height and AGB in northern China and southern China independently (Fig. 4).

3 $$AG{B}_{n}=0.5014\times Lore{y}^{1.8762}$$

4 $$AG{B}_{s}=0.1237\times Lore{y}^{2.3281}$$

where, *AGB*_*n*_ is AGB density (Mg ha^−1^) of field plots in northern China, *AGB*_*s*_ is AGB density of field plots in southern China and Lorey is Lorey’s height. The allometric equation for northern China showed an R^2^ of 0.75 and an RMSE of 35.58 Mg ha^−1^, while for southern China, the R^2^ was 0.50 with an RMSE of 22.96 Mg ha^−1^.

The field data were collected in 2021, therefore they cannot be used to validate the accuracy of a ~2007 AGB map. Given the diverse forest types and vast geographical expanse of China, using these two equations to convert Lorey’s height to forest AGB across China could lead to biases when used in different forest types, and in different areas of China. The fact that the two datasets are very close together does add some confidence that the forest height: biomass relationship of China does not change greatly from location to location. There is precedent in the literature with single equations being used to derive biomass across large regions, with Saatchi, *et al*.^25^ using a single equation per continent in their pantropical biomass map, Baccini, *et al*.^21^ using a single equation across the whole tropics, and the Global Ecosystem Dynamics Investigation (GEDI) LiDAR mission’s biomass product itself using a single equation for all of Asia^33^. In order to assess the impact of this decision, and other errors in the biomass modelling, two independent field datasets collected closer to 2007 from across the woody areas of China were used for validation (Fig. 3).

#### Field data from Zhu, *et al*

One of the datasets consisted of field inventory measurements collected between 2011 and 2016. It included 189 sites with three 20 m × 20 m plots at each site. The longitude and latitude of each site were the averaged values of the three plots within the site (the three plots were usually close to each other, e.g., within 50 m). In order to reduce the uncertainty of biomass change from 2007 to 2016 and the uncertainty of geolocations, 83 field sites were used to validate our map. Field sites were used in analysis if they met the following three criteria^34^.

1. At least half of the area in the buffered region 100 m around the field sites were forests according to the Hansen forest cover data^35^. We created 100 m buffers to reduce the implication of the uncertainty of the geolocations, considering the geolocations of the sites were averaged values of the three plots in each site.
2. In order to show there had been no major changes in the forest in that area between the time of the creation of the maps and the date of the measurement of the field plots, we confirmed that:
  The maximum NDVI of each year calculated from Landsat were stable from 2007 to 2016 (Fluctuations of NDVI less than 0.15).
  The values of ALOS PALSAR and PALSAR-2 HH and HV polarizations were stable from 2007 to 2016 (Fluctuations of HH band and HV band less than 2 in decibel unit).

3) To exclude the field sites that are highly spatial heterogeneous, which may introduce large errors, we removed field sites that have large ratio of standard deviation of the AGB in the 100 m buffers and the field AGB (ratio > 0.25).

#### Field data from National Science & Technology Infrastructure (NSTI)

The other dataset from NSTI (http://www.cnern.org.cn/) was collected from 2005 to 2015 in 29 sites (Fig. 3). There were 5 to 100 plots (10 m × 10 m) in each site. The dataset was collected by nine field research stations, and was measured every few year (normally every five years). We used the average values of the measurements of AGB which were collected around 2007. We refrained from implementing the above filters due to several reasons. Firstly, the geolocation uncertainty of each site varied, making it challenging to apply a uniform filtering approach. Secondly, the size of each field site in this dataset was potentially varied, further complicating the filtering process. In addition, the dataset available for analysis was relatively limited, and applying filters could potentially result in insufficient field sites for validation purposes.

### Satellite data

#### ICESat GLAS Lorey’s height

GLAS which was launched aboard the NASA Ice Cloud and Land Elevation Satellite (ICESat) satellite on January 12, 2003, was the first laser-ranging (LiDAR) instrument for global observations of the Earth. It was designed to measure ice-sheet topography and associated temporal changes, cloud and atmospheric properties, to obtain information on the height and thickness of radiatively important cloud layers, and as a tertiary aim obtain information on tree heights globally.

GLAS provided data with 65 m diameter circular footprints every 170 m along orbits between −86° to 86° latitude. Since we aimed to produce a forest AGB map in ~2007, we selected 287,399 GLAS footprints located on forests^35^ acquired during the operating periods L3H and L3I (Table 2). In this study, we directly used the Lorey’s height values generated by Lefsky^27^, who developed distinct equations for estimating Lorey’s height for needleleaf, broadleaf, and mixed forests globally, based on waveform extent indices from GLAS.

**Table 2:** Reference orbit tracks acquired during ICESat observation periods.

Operation Period	Start Date	End Date	Starting Track	Ending Track
Laser 3H	2007-03-12	2007-04-14	91-day #1279	91-day #426
Laser 3I	2007-10-02	2007-11-05	91-day #1280	91-day #421

**Fig. 5:**
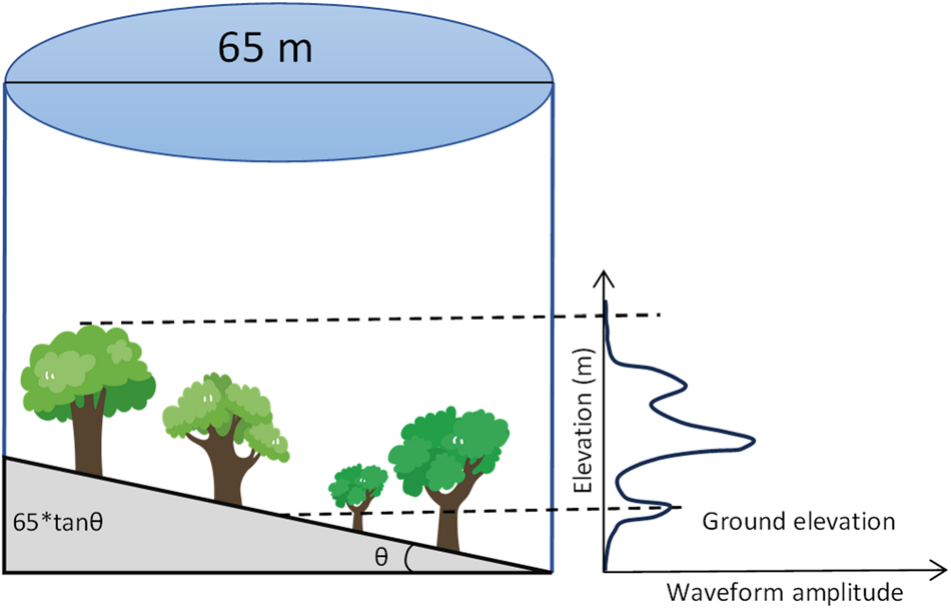
The effects of slope on trees of different heights.

We removed the GLAS footprints over hills with slope > 15°, because of potential overestimates or underestimates in Lorey’s height in high slope areas^28^. We also excluded GLAS footprints in forests with slopes between 12° and 15° and where AGB ≤ 40 Mg ha^−1^ (roughly 11–12 m in height). This is because deriving tree height becomes highly uncertain when the topographic relief within a footprint is large compared to the tree height (Fig. 5), resulting in lower AGB estimates being more susceptible to potential impacts from slope^36^.

#### ALOS PALSAR annual mosaic data

PALSAR was an L-band SAR instrument on ALOS that operated by the Japan Aerospace Exploration Agency (JAXA) between 2006 and 2011, that has proved very useful in mapping forest biomass across a range of ecosystems^37–40^. L-band SAR is the longest wavelength SAR (~24 cm) currently available from space. JAXA produced annual seamless mosaics of the radar backscattered intensity from the PALSAR observations at HH or HV polarization, with terrain and slope correction already applied^41^. We accessed the 2007 mosaic at the resolution of 25 m and did pre-processing through Google Earth Engine (GEE). We applied a focal mean smoothing filter with a radius of 150 m to reduce speckle noise. The digital number (DN) values were converted to gamma naught values in decibel unit (dB) using the equation provided by JAXA:

5 $${\gamma }^{0}=1{0\log }_{10}(D{N}^{2})+CF$$

where, *γ*^0^ is the backscattering coefficient in dB, and CF is a calibration factor equal to −83.0 dB^42^ for the PALSAR mosaic. HV, HH polarizations and the ratio of HV and HH were used in the estimation model, as the polarization ratio has a higher saturation point^43,44^.

#### Envisat ASAR

Envisat was a satellite mission operated by the European Space Agency (ESA). The main objective of the Envisat programme was to study and monitor the Earth’s environment on various scales, from local through regional to global^45^. Envisat was equipped with ten instruments, including an active C-band sensor ASAR. C-band is a shorter wavelength (~6 cm) than L-band, making it suitable for assisting with lower biomass areas; additionally its frequent observations in the Wide Swath mode at a resolution of 150 m allow the generation of the standard deviation of the backscatter through the year, which further helps to understand characteristics of the forests that link to biomass^46^. Envisat ASAR measured the radar backscatter of the Earth’s surface at HH or VV polarization, both of which were collected in our study area.

**Fig. 6:**
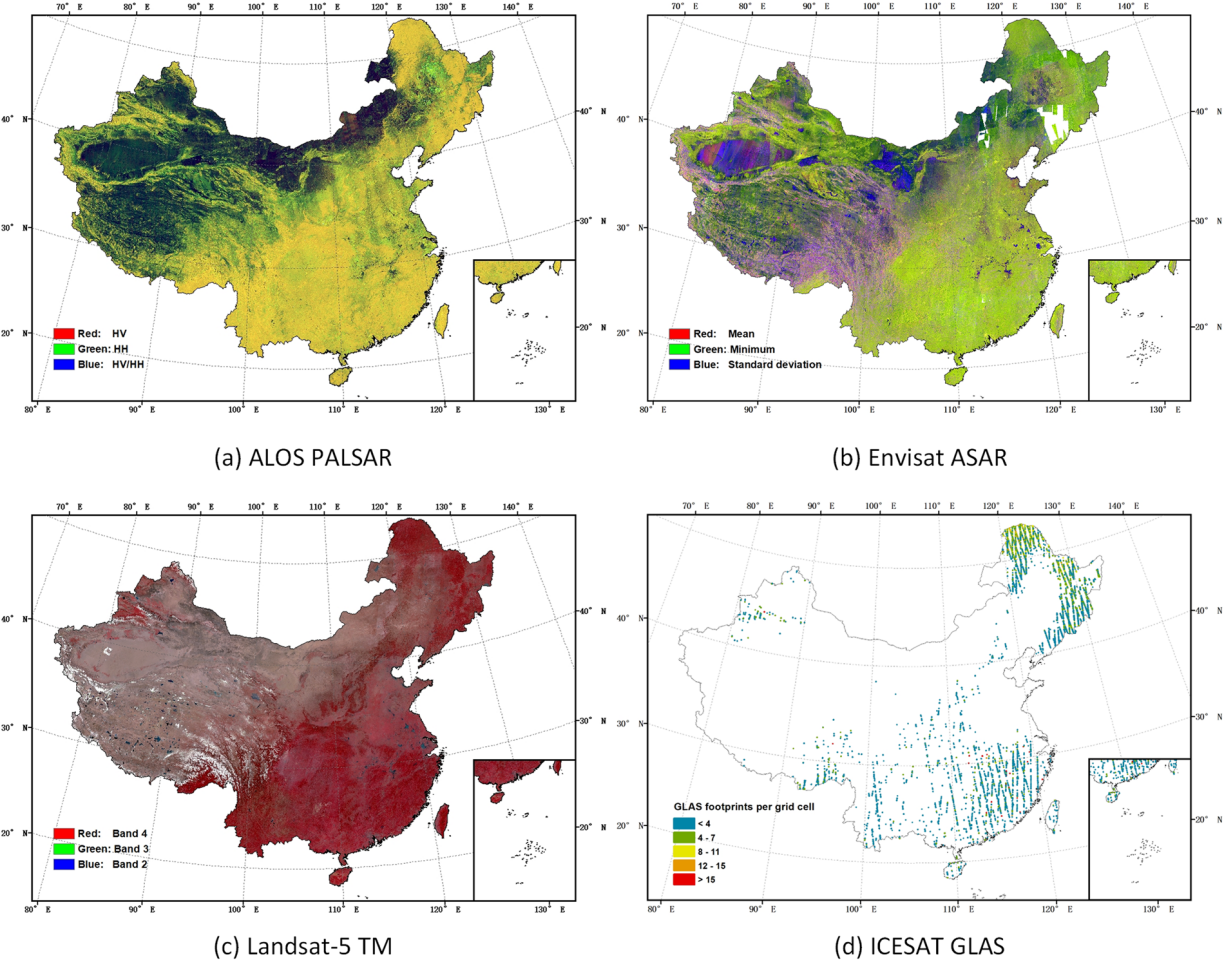
Earth observations used in this study. (**a**) False color composite of ALOS PALSAR annual mosaic data for the year 2007 (HV-polarized backscatter in red, HH-polarized backscatter in green, and the ratio of the backscatter HV/HH in blue). (**b**) False color composite of the Envisat ASAR dataset covering China acquired over 2007 to 2008 (mean of HH and VV in red, minimum of HH and VV in green, and standard deviation of HH and VV in blue). The white areas are where no observations were collected. (**c**) False color composite of temporal mean values computed from Landsat-5 TM bands 2, 3 and 4 acquired from 1st January 2006 to 30th December 2008. Band 4 (Near-infrared) in red, Band 3 (Red) in green, Band 2 (Green) in blue. (**d**) Filtered grid cells of ICESAT GLAS data used to create and test the model.

We calculated the mean values and standard deviation of combined HH and VV images together over 2007 to 2008, as C-band SAR backscatter and forest biomass has similar relationship with both polarizations^47,48^, and this combination reduced artefacts due to differing look angles and number of observations. In order to calculate the mean values and standard deviation, we only used pixels where there were at least two images of each pixel (gaps correspond to 1.2% of the total area of China, see Fig. 6b, where a separate AGB retrieval model not based on Envisat data was used^46^). This method of selecting pixels with sufficient multi-temporal coverage was aimed at ensuring more stable and reliable observations. In total we used 113,698 ASAR images tiled in 1 degree × 1 degree grid cells across China.

#### Landsat-5

Since the first Landsat satellite launched in 1972, Landsat data have contributed to our understanding of Earth, especially in land use and land use change. We used Thematic Mapper (TM) images here, which consist of seven spectral bands with a spatial resolution of 30 meters for Bands 1 to 5 and 7, and a temporal resolution of 16 days. We extracted all the images acquired from 1st January 2006 to 30th December 2008, and used Quality Assessment (QA) band, generated from the CFMASK algorithm^49^, to mask cloud and cloud shadow. We then stacked the cloud-free Landsat-5 bands and computed the mean value of the spectral reflectance in the stack. The maximum, minimum and the differences of maximum and minimum values of Normalized Difference Vegetation Index (NDVI) of each pixel were also calculated because of the strong predictive power when related to AGB^50–53^.

#### Topographic data

NASADEM was generated by reprocessing the SRTM data with other DEM data, such as ICESat GLAS, ASTER GDEM and ALOS PRISM DEM data^54^. NASADEM is more accurate than SRTM, has no holes, and is globally available at the finer resolution of 30 m^55^. In this study, we used the elevation in the NASADEM product as well as terrain slope derived from NASADEM.

### Forest AGB estimation

We converted Lorey’s height derived from GLAS into forest AGB using the allometric equations developed for northern China and southern China independently with the field data. Each individual Lorey’s height estimate from GLAS comes with considerable random errors in height estimation (RMSE was 5.9 m and R^2^ was 0.67^27^), in addition to geographic uncertainty. In order to reduce the influence of these errors, we averaged the Lorey’s height within 0.01 degrees × 0.01 degrees grid cells and obtained 8,981 cells with at least two GLAS footprints (mean 4.14 footprints). We limited Lorey’s height values to 25 m, as our review of the literature showed nearly no plots with Lorey’s height over 25 m in China^56,57^, especially when averaged in 0.01 degrees × 0.01 degrees grid cells.

**Fig. 7:**
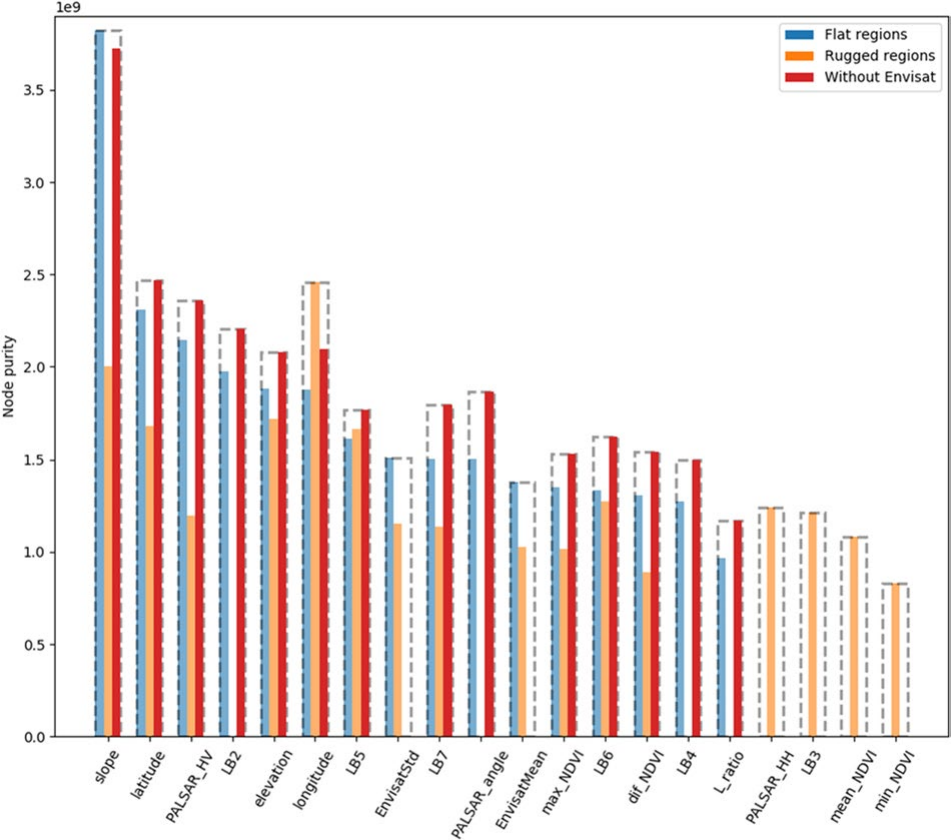
Importance rank of selected variables in RF regression (importance of layers in flat regions from high to low). The six least important variables for all the three regions are not shown in the figure. LBx represents Landsat-5 Band x, dif_NDVI represents the differences of maximum and minimum values of NDVI. EnvisatMean and EnvisatStd represent the mean values and standard deviation of Envisat data.

All remote sensing imagery was resampled to a spatial resolution of 50 meters in GEE. We then used Random Forest (RF) regression^58^ to extrapolate AGB grid cells from GLAS data to a continuous map at 50 m resolution. We trained the RF regression using the variables derived from Earth Observation datasets listed above as well as layers giving latitude and longitude, to enable the model to take account of the differing relationships across the varied ecosystems of China. As we were focused only on forest AGB, we used the 30 m resolution Hansen *et al*. forest cover data^35^ to mask all the input layers to forest only, preventing any influence of non-forest areas on the RF regression. The 2007 forest cover map was obtained by removing pixels flagged as lost between 2000 and 2007, from baseline forest canopy cover >10% in the year 2000 canopy cover layer. However, due to the lack of forest gains between 2000 and 2007, the 2007 forest cover map may underestimate the actual extent of forest cover. Since the importance of the variables differs in flat region and rugged region (Fig. 7), we modelled the AGB in flat region and rugged region separately to make full use of the remote sensing layers.

We eliminated redundant information and thus improved the computational efficiency of prediction by analysing the importance of all input layers on the AGB map. We considered the node purity, which is equivalent to Mean Decrease Gini, of each variable (Fig. 7). The higher the value of node purity, the higher the importance of the variable to our model. This is because node purity increased along with a decrease in the residual sum of squares, indicating a decrease in the Gini coefficient in regression analysis^59,60^. We removed the 10 variables with smallest node purity in each of the three models.

### Accuracy and bias correction

To evaluate the performance of the AGB estimation model, 8 981 grid cells of 0.01 degrees × 0.01 degrees AGB were randomly split in training set (60% of the data) and validation set (40% of the data). The performance of the model was assessed by aggregating the pixel-wise AGB estimates to the same size of grid cells at 0.01 degrees × 0.01 degrees. The coefficient of determination (R^2^) and Root Mean Square Error (RMSE) were used to quantify the performance of the model.

**Fig. 8:**
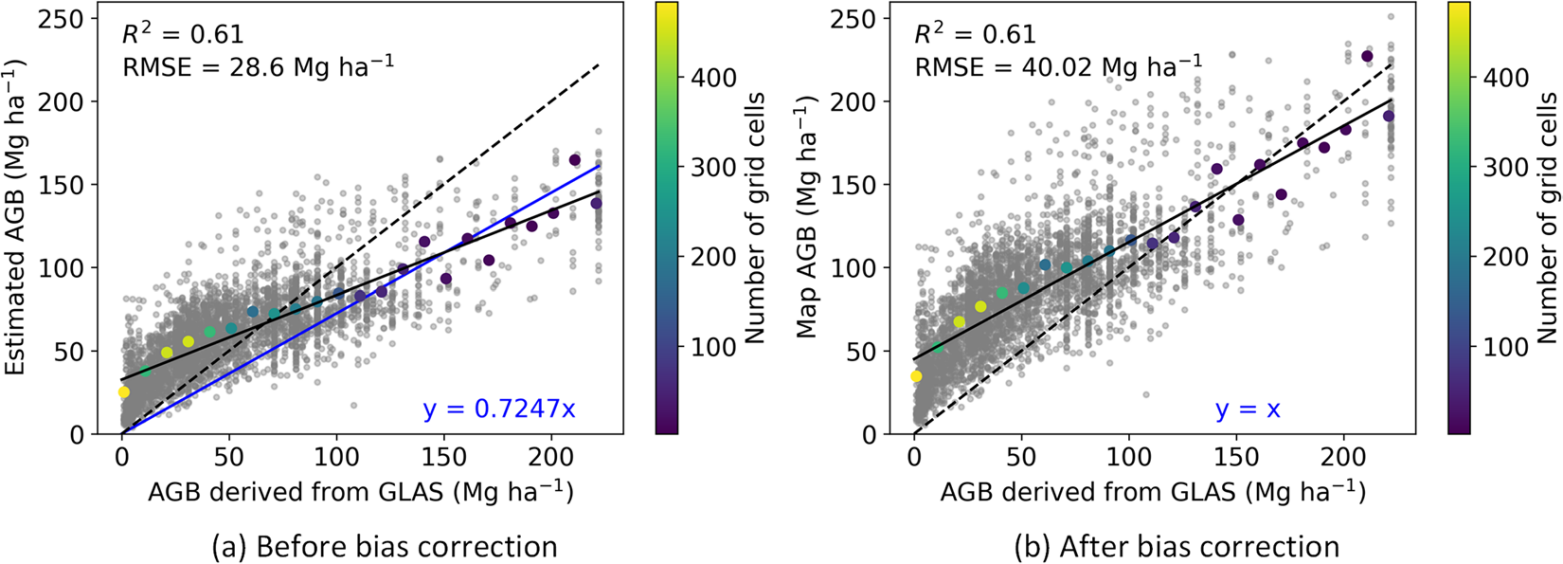
Evaluation of the model. (**a**) Estimated AGB from our model against AGB of averaged grid cells derived from GLAS data (grey points). (**b**) Bias corrected AGB against AGB of averaged grid cells derived from GLAS data (grey points). The dotted line is the 1:1 line. The black solid line is the regression of the whole set of points. The colour dots are the average values of 10 Mg ha^–1^ interval of AGB derived from GLAS, and the colour bar represents the number of grid cells in a given AGB interval. The blue line is a standard regression of colour dots with the intercept forced to be zero; in the bias corrected figure (**b**) this line overlaps exactly with the 1:1 line and is thus not visible.

The model estimated AGB corresponded well with the AGB derived from GLAS, and the R^2^ and RMSE between the estimated AGB and AGB derived from GLAS were 0.61 and 28.6 Mg ha^−1^, respectively (Fig. 8a). Our method tended to overestimate forest AGB at the forests with low AGB (AGB <60 Mg ha^−1^), and underestimate in forests with large AGB, as is common with RF models. To correct for this bias, a linear regression bias correction was performed to the estimated AGB using the slope of the fitting line of 10 Mg ha^−1^ intervals^61–63^. This improved, but did not solve, the bias issue, with increased scatter in the output (RMSE = 40 Mg ha^−1^ compared to 28.6 Mg ha^−1^ pre-correction), but with the range of values better matching the range from the GLAS predictions (maximum predicted value 251 Mg ha^−1^ after correction, compared to 182 Mg ha^−1^ pre-correction, compared to the maximum GLAS prediction of 222 Mg ha^−1^).

## Data Records

The new forest AGB map was produced using multisource remote sensing datasets, including ICESat GLAS data acquired in 2007, ALOS PALSAR data acquired in 2007, Envisat ASAR data acquired in 2007 and 2008, Landsat-5 data acquired from 2006 to 2008, and NASADEM data. Due to the remote sensing datasets all acquired around 2007, it is a circa 2007 forest AGB map. The AGB map is stored in GeoTIFF (.tif) files on Edinburgh DataShare^64^. The map provides the values of AGB in Mg ha^−1^, and the values have been rounded to integers. It can be converted to carbon by multiplying by 0.47^65^.

## Technical Validation

Our estimates of total carbon stored in forest AGB in China is 9.52 Pg C. To assess the accuracy of our AGB map, we validated our map using one field datasets from a previous study^34^ and the other independent dataset from NSTI. The coefficient of determination (R^2^) and Root Mean Square Error (RMSE) were used to quantify the performance of the maps. In addition, we compared this map to the GEOCARBON map^6^, Su map^23^ and CCI map^9^ and attempt to explain the differences.

### Validation using field data

#### Comparison to Field data from Zhu, *et al*

**Fig. 9:**
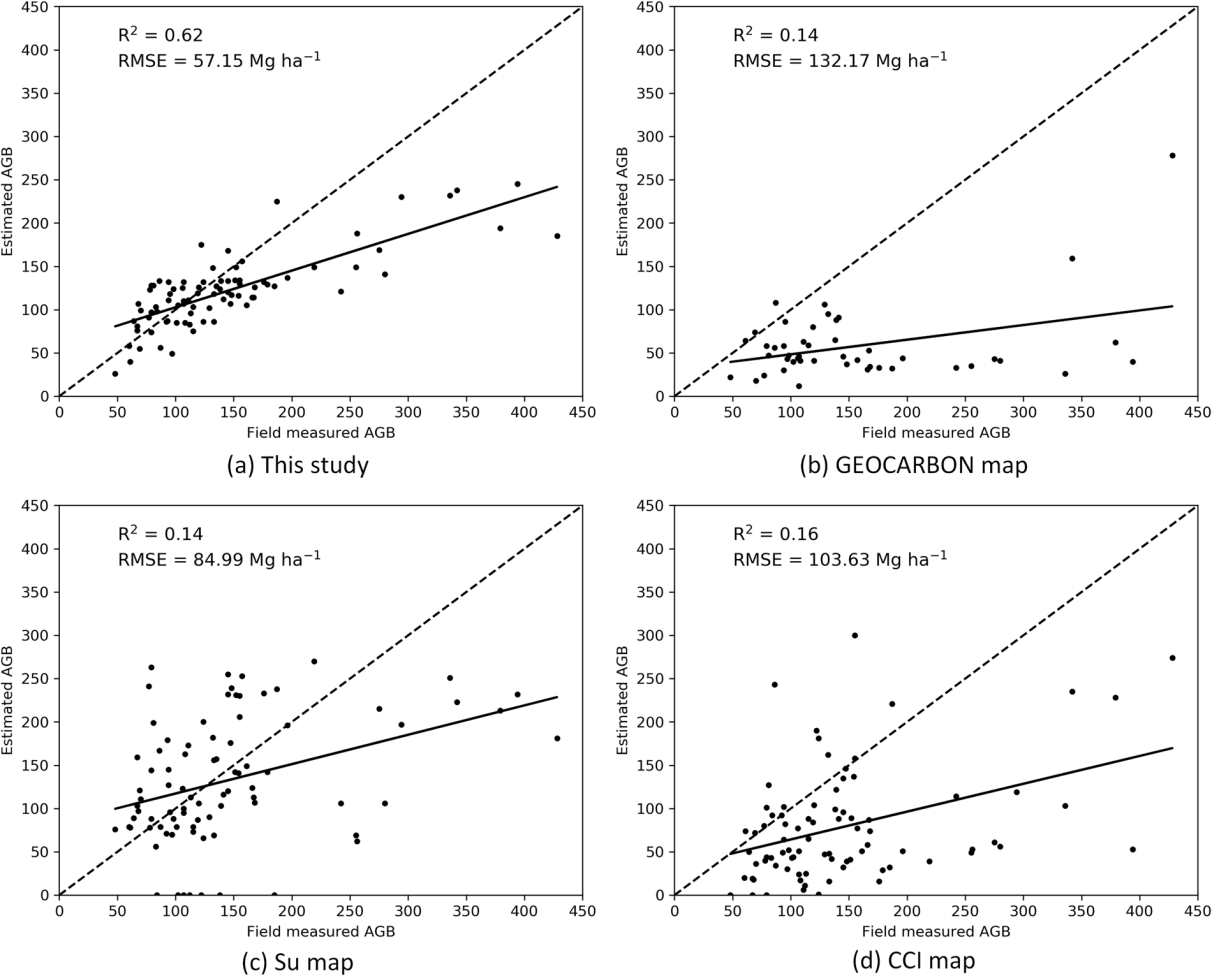
Assessment of AGB map against independent field data from Zhu, *et al*. The dotted line is the 1:1 line.

We used 83 sites from Zhu, *et al*.^34^ to validate our map at a pixel level. Overall, the estimated AGB of our map demonstrated a reasonably good correspondence with field measured AGB, with an R^2^ of 0.62 and an RMSE of 57.15 Mg ha^−1^; this exceeds considerably the metrics obtained from the other datasets (Fig. 9). Compared with the field data, all the four maps exhibited a tendency to underestimate AGB values in areas with large AGB (>200 Mg ha^−1^). The other national scale AGB map, the Su map^23^, exhibited a lower RMSE than the two global AGB maps.

**Fig. 10:**
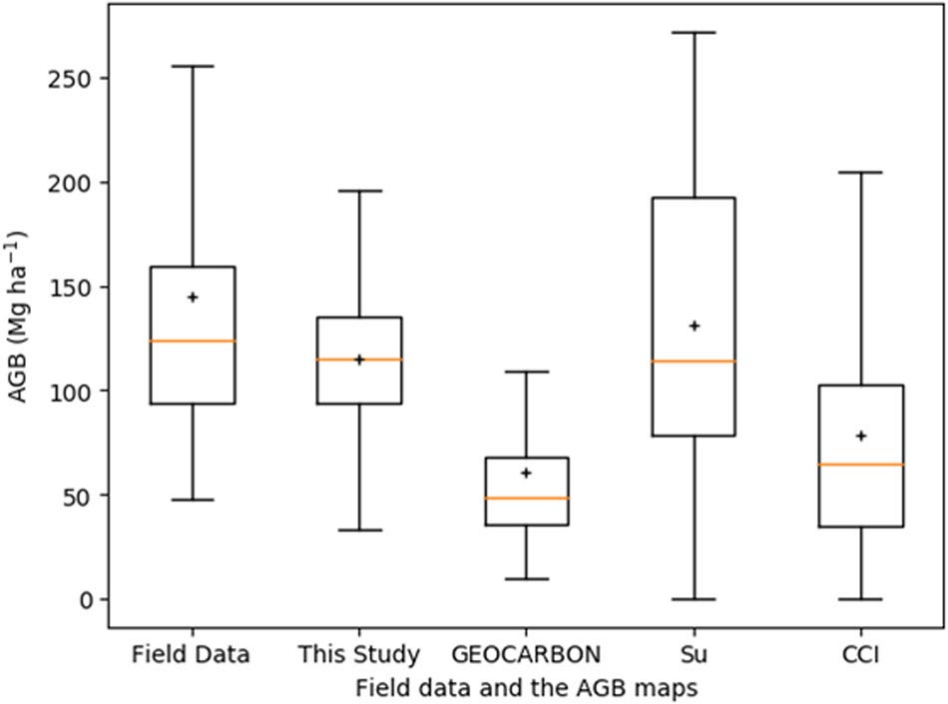
Boxplot of field data and forest AGB of the four AGB maps within 1 km × 1 km grid cells of field sites. The orange horizontal bar indicates the median and the boxes show the interquartile range, and the “+” indicates the mean forest AGB.

To assess the similarities and differences in the AGB distribution, we compared field data from Zhu, *et al*.^34^ and the pixel values of the four AGB maps within 1 km × 1 km grid cells, which represents the resolution of the coarsest map (Fig. 10). The field data hosted the highest median and mean AGB, and a half of the field sites had an AGB density of 95–160 Mg ha^−1^. The map from this study had a similar low end of the interquartile range with the field data, but was more homogeneously distributed with 75% of the AGB below 130 Mg ha^−1^. GEOCARBON map hosted the smallest median and mean AGB and was most homogeneously distributed. The AGB of Su map had the largest dispersion, with 50% of the AGB were between 80 Mg ha^−1^ and 190 Mg ha^−1^. The CCI map had the second smallest median AGB of 60 Mg ha^−1^. It can be seen that the two national scale AGB maps are closer match to these field data in this comparison.

#### Comparison to Field data from NSTI

**Fig. 11:**
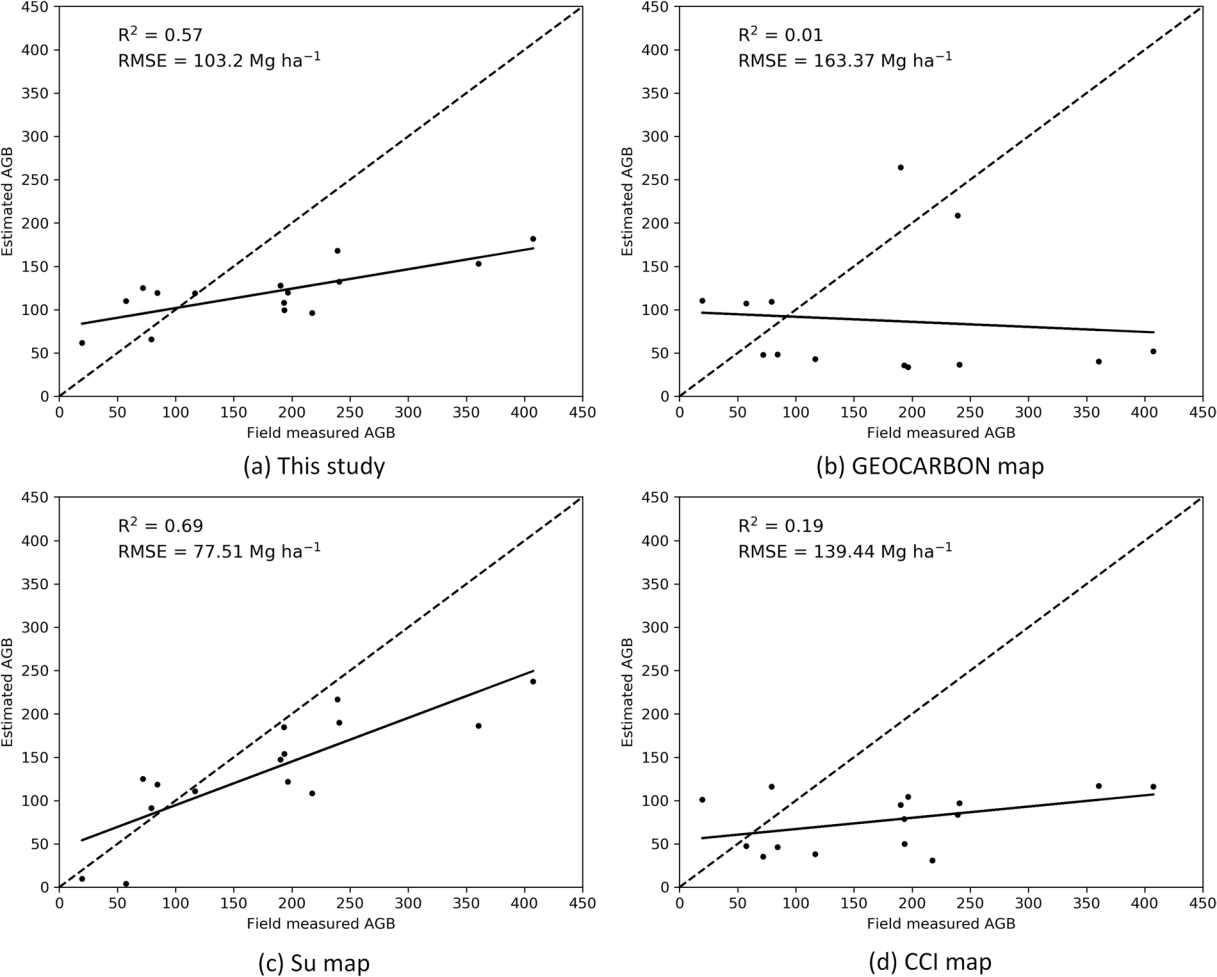
Assessment of AGB map against independent field data from NSTI. The dotted line is the 1:1 line.

We also validated our map, and the GEOCARBON, Su and CCI maps using field data from NSTI. The uncertainty of the geolocations of the data from NSTI were from 0.0001 degrees to 0.01 degrees (~10 m–1000 m). In order to reduce the effect of errors caused by the coarse geolocation, we aggregated our estimated forest AGB map and the field data to 0.01 degrees × 0.01 degrees grid cells. 15 grid cells aggregated from 29 field sites were used to validate the four AGB maps. The Su map had the strongest correlation with the field data from NSTI, which can be attributed to the incorporation of part of the field data in their training dataset (so this is not an independent test of the Su map). Our map demonstrated the second best correlation with this field data, with an R^2^ of 0.57 and an RMSE of 103.2 Mg ha^−1^. All four maps showed relatively high RMSE values when compared to the field data (Fig.11). This can be attributed to the limited coverage of field measurements within the 0.01 × 0.01 degrees grid cell. In fact, even within the grid cell with the highest number of field plots, the area of the field measurements cover ~1% of the total area of the grid cell.

**Fig. 12:**
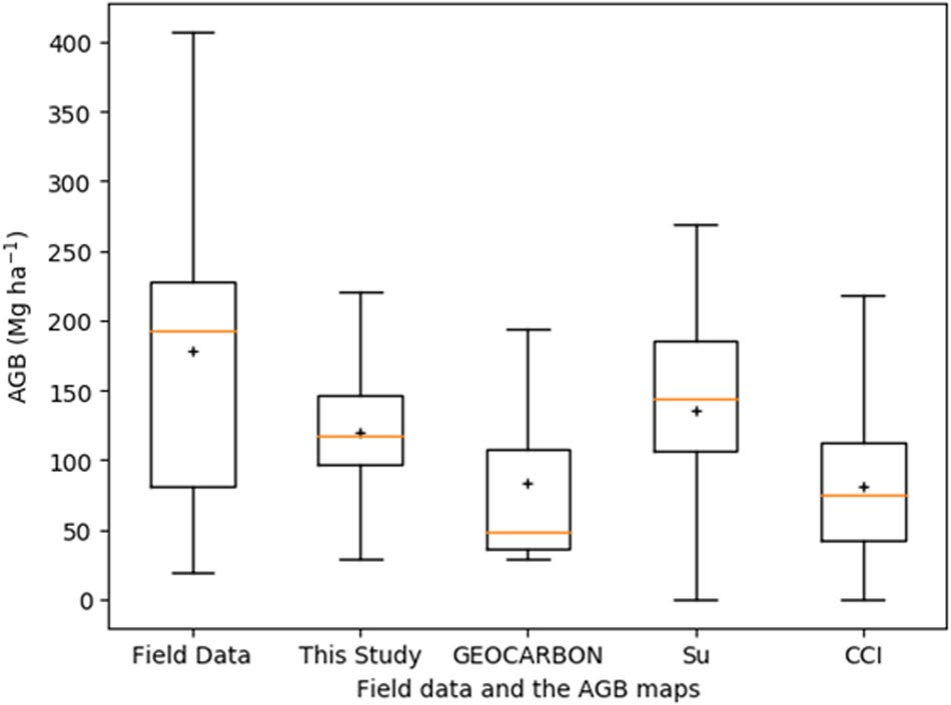
Boxplot of field data and forest AGB of the four AGB maps within 1 km × 1 km grid cells of field sites. The orange horizontal bar indicates the median and the boxes show the interquartile range, and the “+” indicates the mean forest AGB.

The field data from NSTI had a large range of forest AGB, with 50% of the field data between 80 Mg ha^−1^ and 230 Mg ha^−1^ (Fig. 12). The field data had the largest median and mean AGB. 50% of our map had an AGB of 100–150 Mg ha^−1^, and both of median and mean AGB were around 120 Mg ha^−1^. Both of GEOCARBON map and CCI map had 50% AGB between 40 Mg ha^−1^ and 100 Mg ha^−1^. The two maps had similar mean AGB of 80 Mg ha^−1^, but the median AGB of GEOCARBON map is lower. The Su map had the second largest median AGB and mean AGB of 140 Mg ha^−1^and 130 Mg ha^−1^, respectively.

### Comparison to existing AGB maps

**Fig. 13:**
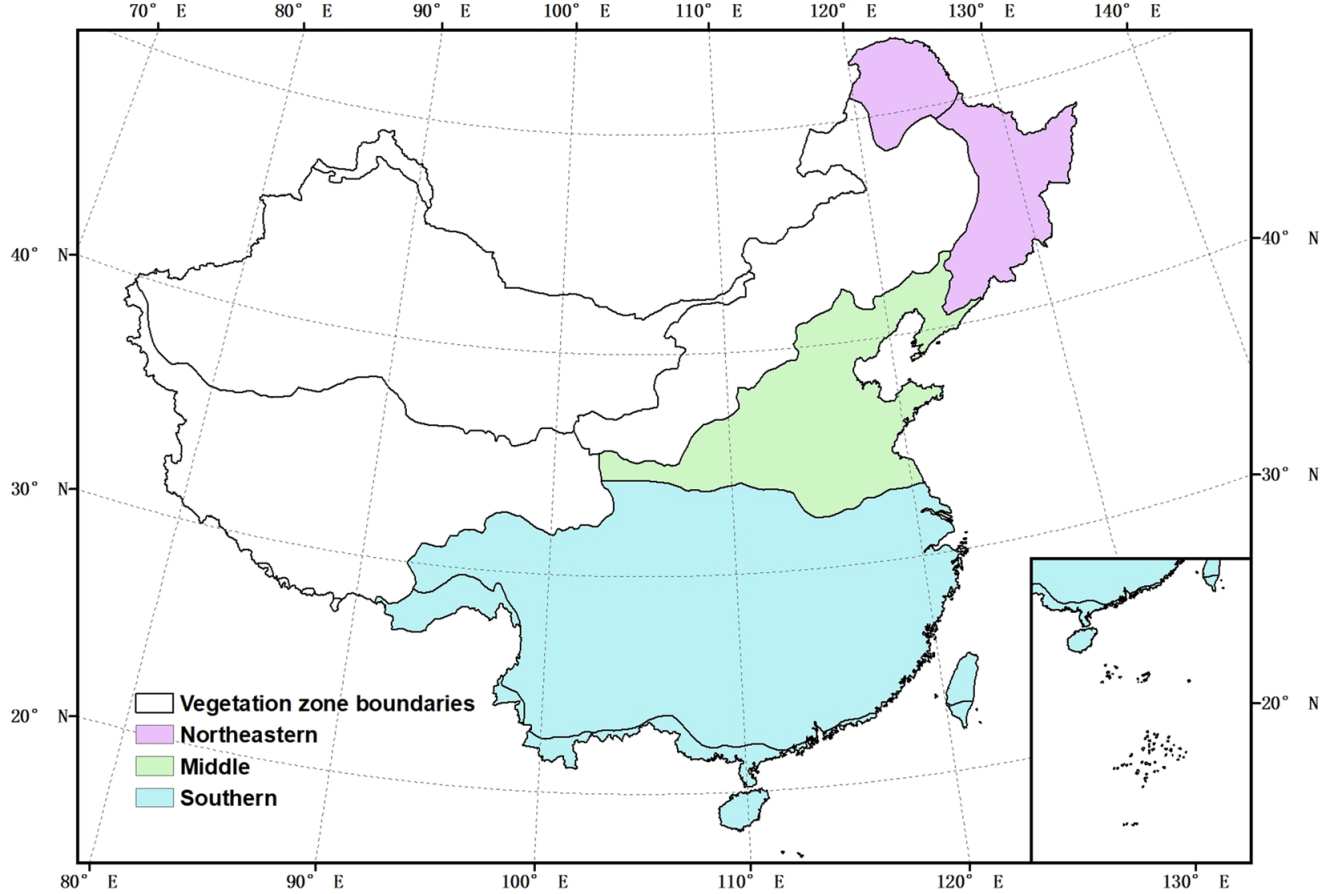
Forest zones we used to compare the forest AGB maps.

The bias corrected AGB map from this study was compared with AGB maps by Avitabile, *et al*.^6^, Su, *et al*.^23^, and Santoro and Cartus^9^. Some of the maps are generated globally, while others are for specific smaller regions. We masked the non-forest area of Santoro and Cartus^9^ using the Hansen forest cover map in 2007^35^, as this map expresses woody biomass and we focused on forest AGB in this study. In order to compare the maps at pixel level, we reprojected and resampled the AGB maps to the same projection and resolution as our map using a nearest neighbour resampling method, avoiding uncertainty which can be introduced during warping, and appropriate as our map is much higher resolution than the maps compared. Forest AGB density were converted to above-ground carbon using a default conversion factor of 0.47^65^. We compared the AGB maps across China and for three specific regions: southern, northeastern and middle part of China (Fig. 13). These regions were chosen due to their extensive forest coverage and were derived by combining small vegetation zones^66^. Compared to the three AGB maps, our results show large differences in total carbon and the distribution of AGB, in particular in southern China, where there are dense forest and steep slopes which introduce large uncertainties in AGB estimation.

#### Across China

**Fig. 14:**
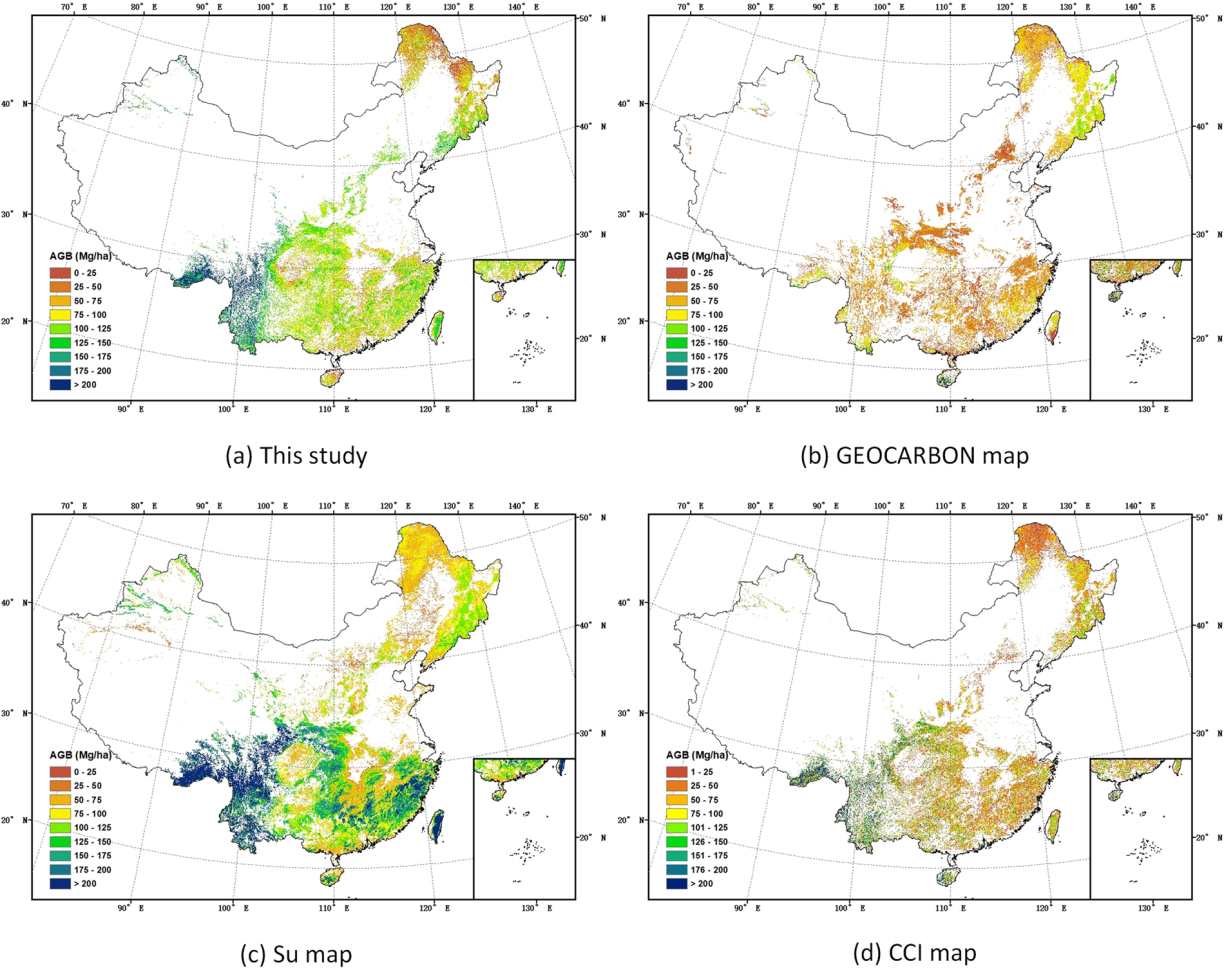
Spatial distribution of forest AGB across China.

All four forest AGB maps showed considerable higher AGB in southwestern China, and the lowest AGB in northern China. However, significant differences were visible when the four AGB maps were compared (Fig. 14).

Among the four AGB maps, average AGB from the Su map^23^ was higher than the other three maps, while the average AGB from this study was the second highest and differed greatly from the values from GEOCARBON map^6^ and CCI Biomass map^9^.

Some of the differences in average AGB and total carbon storage in Table 3, may relate to the differences in the total area of forest used in the calculations. According to assessment report by the Food and Agriculture Organization (FAO), the forest area in China was 193.0 million ha in 2005^67^. The forest area from this study, which used the Hansen forest cover map^35^ to mask the non-forest area, was close to the forest area by FAO. The explanation for a smaller forest area from CCI map^9^, which we also used the Hansen forest cover map^35^ to mask non-forest area, was that we only counted the pixel values higher than zero as forest, leading a small difference in the forest area of the two maps. The GEOCARBON map^6^ and Su map^23^ showed significant differences in the estimated forest area compared to this study. This is because the two AGB maps used different forest cover maps, the GLC2000 map^68^ and the 2000 land use map^69^. The total above-ground carbon stocks for China were estimated by summing the carbon of all pixels in each AGB map. The total carbon stored in China’s forests was estimated 9.52 Pg C in this study, which was almost two and a half times as much as that in GEOCARBON map^6^, 60% higher than that in CCI map^9^, but less than 60% of that in the Su map^23^ (Table 3).

**Table 3:** Average AGB, forest area and total carbon in China.

Maps	Year	Resolution	Average AGB (Mg ha^−1^)	Forest area (10^6 ^ha)	Total carbon (Pg C)
This study	~2007	50 m	104.10	194.53	9.52
GEOCARBON map	~2000–2010	0.01 degrees (~1000 m)	53.10	157.03	3.92
Su map	2004	1000 m	121.93	297.03	17.02
CCI map	2010	100 m	73.77	171.24	5.94

**Fig. 15:**
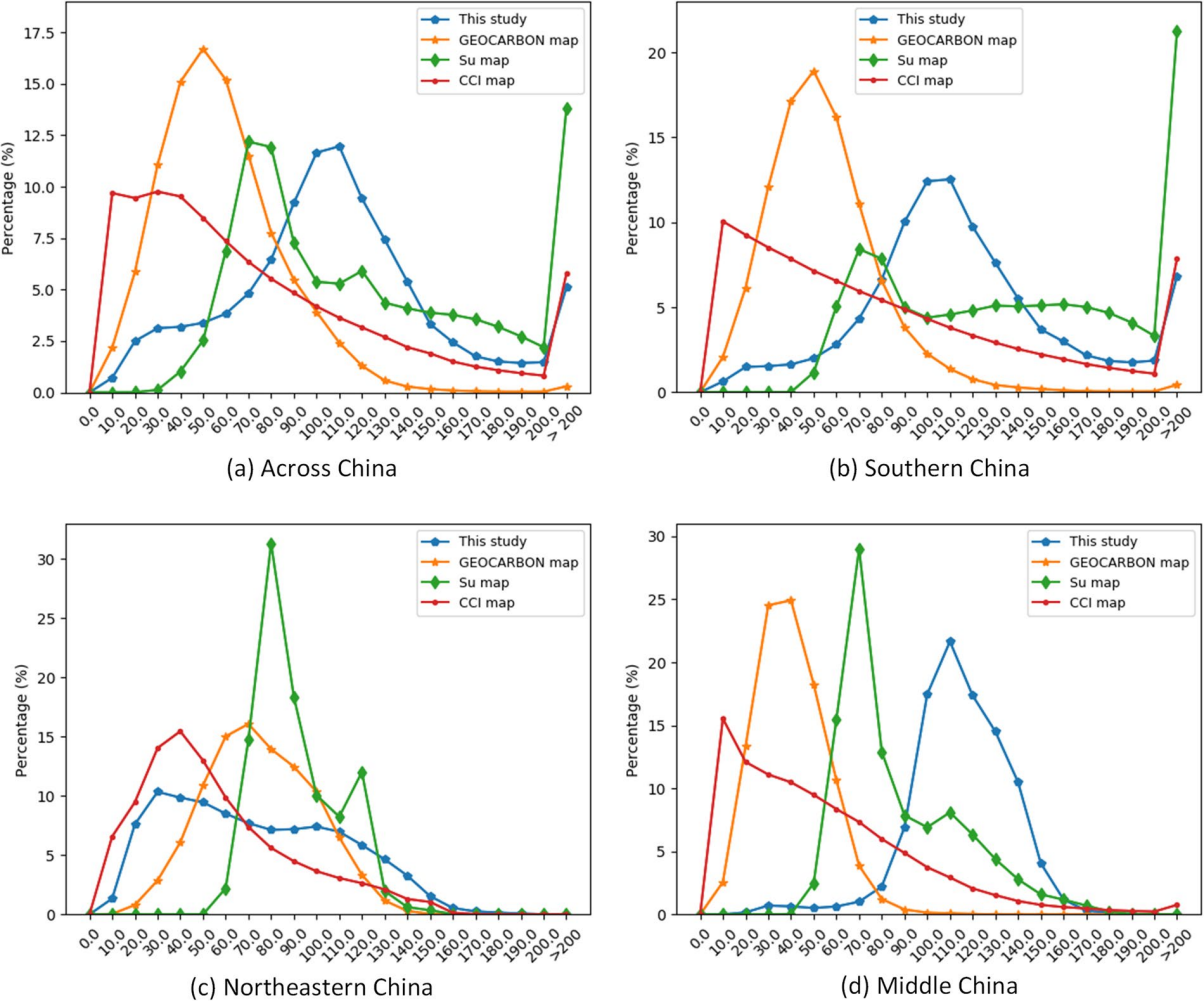
Histogram of AGB in 10 Mg ha^−1^ bins, 10 indicts 0–10 bin.

The histograms of the four AGB maps across China in 10 Mg ha^−1^ bins showed that the peak of frequency of AGB in CCI Biomass^9^ occurred at a lower AGB of 0 to 40 Mg ha^−1^, while the peak value in Su map was the highest (Fig. 15). The frequency of AGB in this study peaked at 100 to 110 Mg ha^−1^, and more than half of the AGB ranged from 70 to 130 Mg ha^−1^. More than 80% of AGB in GEOCARBON map^6^ ranged from 20 to 90 Mg ha^−1^, with nearly no AGB higher than 150 Mg ha^−1^. The AGB in CCI Biomass map^9^ had no AGB in 10 Mg ha^−1^ bins greater than 10%, and the frequency of AGB decreased from 40 Mg ha^−1^, and increased at the >200 Mg ha^−1^ interval. The AGB histogram for southern China is similar to that of the entire country, with the exception that the Su map contains a greater frequency of AGB values exceeding 200 Mg ha^−1^. In the northeastern and middle China, the AGB is predominantly less than 150 Mg ha^−1^.

Based on the assessment of the field data above and comparisons with existing AGB maps, we would recommend that this new dataset represents the best estimate of the total carbon storage, and its distribution, for China in this time period.

## Data Availability

The code used to process Envisat ASAR data is written in IDL. The code for processing remote sensing data and producing continuous AGB map is written in Google Earth Engine using JavaScript. All the code is available at: https://github.com/uoedwq/ForestAGB.

## References

[1] Pörtner, H.-O. *et al*. *Climate change 2022: Impacts, adaptation and vulnerability*. (IPCC Geneva, Switzerland:, 2022).

[2] Mitchard ET (2018). The tropical forest carbon cycle and climate change. Nature.

[3] Grace J, Mitchard E, Gloor E (2014). Perturbations in the carbon budget of the tropics. Global Change Biology.

[4] McDowell NG (2020). Pervasive shifts in forest dynamics in a changing world. Science.

[5] Besnard S (2021). Global sensitivities of forest carbon changes to environmental conditions. Global Change Biology.

[6] Avitabile, V. *et al*. in *International Conference GV2M, Avignon, France*. 251-252.

[7] Yang L, Liang S, Zhang Y (2020). A new method for generating a global forest aboveground biomass map from multiple high-level satellite products and ancillary information. IEEE Journal of Selected Topics in Applied Earth Observations and Remote Sensing.

[8] Spawn SA, Sullivan CC, Lark TJ, Gibbs HK (2020). Harmonized global maps of above and belowground biomass carbon density in the year 2010. Scientific Data.

[9] Santoro, M. & Cartus, O. ESA biomass climate change initiative (Biomass_cci): Global datasets of forest above-ground biomass for the years 2010, 2017 and 2018, v3. *Cent. Environ. Data Anal* (2021).

[10] Mitchard ET (2014). Markedly divergent estimates of A mazon forest carbon density from ground plots and satellites. Global ecology and biogeography.

[11] Santoro M (2021). The global forest above-ground biomass pool for 2010 estimated from high-resolution satellite observations. Earth System Science Data.

[12] Rodríguez-Veiga P (2019). Forest biomass retrieval approaches from earth observation in different biomes. International Journal of Applied Earth Observation and Geoinformation.

[13] FAO. Global Forest Resources Assessment 2020: Main report. Rome. (2020).

[14] Fang J, Chen A, Peng C, Zhao S, Ci L (2001). Changes in forest biomass carbon storage in China between 1949 and 1998. Science.

[15] Wenhua L (2004). Degradation and restoration of forest ecosystems in China. Forest Ecology and Management.

[16] Viña A, McConnell WJ, Yang H, Xu Z, Liu J (2016). Effects of conservation policy on China’s forest recovery. Science advances.

[17] Chave J (2004). Error propagation and scaling for tropical forest biomass estimates. Philosophical Transactions of the Royal Society of London. Series B: Biological Sciences.

[18] Henry M (2011). Estimating tree biomass of sub-Saharan African forests: a review of available allometric equations. Silva Fennica.

[19] Blackard J (2008). Mapping US forest biomass using nationwide forest inventory data and moderate resolution information. Remote sensing of Environment.

[20] Mitchard, E. T. *et al*. Using satellite radar backscatter to predict above‐ground woody biomass: A consistent relationship across four different African landscapes. *Geophysical Research Letters* **36** (2009).

[21] Baccini A (2012). Estimated carbon dioxide emissions from tropical deforestation improved by carbon-density maps. Nature climate change.

[22] Araza A (2022). A comprehensive framework for assessing the accuracy and uncertainty of global above-ground biomass maps. Remote Sensing of Environment.

[23] Su Y (2016). Spatial distribution of forest aboveground biomass in China: Estimation through combination of spaceborne lidar, optical imagery, and forest inventory data. Remote Sensing of Environment.

[24] Liu J, Liang M, Li L, Long H, De Jong W (2017). Comparative study of the forest transition pathways of nine Asia-Pacific countries. Forest Policy and Economics.

[25] Saatchi SS (2011). Benchmark map of forest carbon stocks in tropical regions across three continents. Proceedings of the national academy of sciences.

[26] Naidoo L (2015). Savannah woody structure modelling and mapping using multi-frequency (X-, C-and L-band) Synthetic Aperture Radar data. ISPRS Journal of Photogrammetry and Remote Sensing.

[27] Lefsky, M. A. A global forest canopy height map from the Moderate Resolution Imaging Spectroradiometer and the Geoscience Laser Altimeter System. *Geophysical Research Letters* **37** (2010).

[28] Chen Q (2010). Retrieving vegetation height of forests and woodlands over mountainous areas in the Pacific Coast region using satellite laser altimetry. Remote Sensing of Environment.

[29] Jucker T (2018). Topography shapes the structure, composition and function of tropical forest landscapes. Ecology letters.

[30] Luo Y (2019). ChinAllomeTree 1.0: China’s normalized tree biomass equation dataset. Earth System Science Data Discussions.

[31] Liu K, Shen X, Cao L, Wang G, Cao F (2018). Estimating forest structural attributes using UAV-LiDAR data in Ginkgo plantations. ISPRS journal of photogrammetry and remote sensing.

[32] Cao L (2016). Estimation of forest biomass dynamics in subtropical forests using multi-temporal airborne LiDAR data. Remote Sensing of Environment.

[33] Duncanson L (2022). Aboveground biomass density models for NASA’s Global Ecosystem Dynamics Investigation (GEDI) lidar mission. Remote Sensing of Environment.

[34] Zhu J (2017). Carbon stocks and changes of dead organic matter in China’s forests. Nature Communications.

[35] Hansen MC (2013). High-resolution global maps of 21st-century forest cover change. science.

[36] Harding, D. J. & Carabajal, C. C. ICESat waveform measurements of within‐footprint topographic relief and vegetation vertical structure. *Geophysical research letters* **32** (2005).

[37] Hamdan O, Aziz HK, Hasmadi IM, L-band ALOS (2014). PALSAR for biomass estimation of Matang Mangroves, Malaysia. Remote Sensing of Environment.

[38] Carreiras JM, Vasconcelos MJ, Lucas RM (2012). Understanding the relationship between aboveground biomass and ALOS PALSAR data in the forests of Guinea-Bissau (West Africa). Remote Sensing of Environment.

[39] Bouvet A (2018). An above-ground biomass map of African savannahs and woodlands at 25 m resolution derived from ALOS PALSAR. Remote sensing of environment.

[40] Chen L, Wang Y, Ren C, Zhang B, Wang Z (2019). Assessment of multi-wavelength SAR and multispectral instrument data for forest aboveground biomass mapping using random forest kriging. Forest ecology and management.

[41] Shimada M (2014). New global forest/non-forest maps from ALOS PALSAR data (2007–2010). Remote Sensing of environment.

[42] Motohka T, Shimada M, Uryu Y, Setiabudi B (2014). Using time series PALSAR gamma nought mosaics for automatic detection of tropical deforestation: A test study in Riau, Indonesia. Remote Sensing of Environment.

[43] Sarker MLR, Nichol J, Ahmad B, Busu I, Rahman AA (2012). Potential of texture measurements of two-date dual polarization PALSAR data for the improvement of forest biomass estimation. ISPRS Journal of Photogrammetry and Remote Sensing.

[44] Hayashi M, Motohka T, Sawada Y (2019). Aboveground biomass mapping using alos-2/palsar-2 time-series images for borneo’s forest. IEEE Journal of Selected Topics in Applied Earth Observations and Remote Sensing.

[45] Louet, J. & Bruzzi, S. in *IEEE 1999 International Geoscience and Remote Sensing Symposium. IGARSS'99 (Cat. No. 99CH36293)*. 1680–1682 (IEEE).

[46] Santoro M (2015). Strengths and weaknesses of multi-year Envisat ASAR backscatter measurements to map permanent open water bodies at global scale. Remote Sensing of Environment.

[47] Santoro M (2015). Forest growing stock volume of the northern hemisphere: Spatially explicit estimates for 2010 derived from Envisat ASAR. Remote Sensing of Environment.

[48] Castel T (2001). Sensitivity of space-borne SAR data to forest parameters over sloping terrain. Theory and experiment. International journal of remote sensing.

[49] Foga S (2017). Cloud detection algorithm comparison and validation for operational Landsat data products. Remote sensing of environment.

[50] Piao, S., Fang, J., Zhu, B. & Tan, K. Forest biomass carbon stocks in China over the past 2 decades: Estimation based on integrated inventory and satellite data. *Journal of Geophysical Research: Biogeosciences* **110** (2005).

[51] Dong J (2003). Remote sensing estimates of boreal and temperate forest woody biomass: carbon pools, sources, and sinks. Remote sensing of Environment.

[52] Saatchi SS, Houghton RA, Dos Santos Alvala R, Soares JV, Yu Y (2007). Distribution of aboveground live biomass in the Amazon basin. Global change biology.

[53] Lumbierres M, Méndez PF, Bustamante J, Soriguer R, Santamaría L (2017). Modeling biomass production in seasonal wetlands using MODIS NDVI land surface phenology. Remote Sensing.

[54] Crippen, R. *et al*. NASADEM global elevation model: methods and progress. (2016).

[55] Uuemaa E, Ahi S, Montibeller B, Muru M, Kmoch A (2020). Vertical accuracy of freely available global digital elevation models (ASTER, AW3D30, MERIT, TanDEM-X, SRTM, and NASADEM). Remote Sensing.

[56] Huang H, Liu C, Wang X, Zhou X, Gong P (2019). Integration of multi-resource remotely sensed data and allometric models for forest aboveground biomass estimation in China. Remote sensing of environment.

[57] Luo Y, Zhang X, Wang X, Lu F (2014). Biomass and its allocation of Chinese forest ecosystems: Ecological Archives E095-177. Ecology.

[58] Breiman L (2001). Random forests. Machine learning.

[59] Han, H., Guo, X. & Yu, H. in *2016 7th ieee international conference on software engineering and service science (icsess)*. 219–224 (IEEE).

[60] Wang Y (2019). A random forest model to predict heatstroke occurrence for heatwave in China. Science of the Total Environment.

[61] Song J (2015). Bias corrections for Random Forest in regression using residual rotation. Journal of the Korean Statistical Society.

[62] Shendryk Y (2022). Fusing GEDI with earth observation data for large area aboveground biomass mapping. International Journal of Applied Earth Observation and Geoinformation.

[63] Liang M, Duncanson L, Silva JA, Sedano F (2023). Quantifying aboveground biomass dynamics from charcoal degradation in Mozambique using GEDI Lidar and Landsat. Remote Sensing of Environment.

[64] Dong W, Mitchard ETA, Santoro M, Chen M, Wheeler CE (2023). University of Edinburgh. School of GeoSciences..

[65] IPCC. 2006 Intergovernmental Panel on Climate Change (IPCC) Guidelines for National Greenhouse Gas Inventories, Vol. 4: Agriculture, Forestry and Other Land Use, Institute for Global Environmental Strategies (IGES). *Hayama, Japan on behalf of the IPCC, 2006* (2006).

[66] Hou, X. Vegetation atlas of China. *Chinese Academy of Science, the editorial board of vegetation map of China*, 113–124 (2001).

[67] FAO. Global Forest Resources Assessment 2010 - Country Report: China. FRA 2010/042, FAO, Rome. (2010).

[68] Bartholome E, Belward AS (2005). GLC2000: a new approach to global land cover mapping from Earth observation data. International Journal of Remote Sensing.

[69] Liu J (2002). The land use and land cover change database and its relative studies in China. Journal of Geographical Sciences.

[70] Yin G (2015). MODIS Based Estimation of Forest Aboveground Biomass in China. PLOS ONE.

[71] Li N (2015). Biomass Resources Distribution in the Terrestrial Ecosystem of China. Sustainability.

